# SARS-CoV-2 infection and COVID-19 and human reproduction – A changing perspective – A 2022 update

**DOI:** 10.1016/j.clinsp.2023.100166

**Published:** 2023-01-24

**Authors:** Luciana C. Delamuta, Pedro A.A. Monteleone, Edson S. Ferreira-Filho, Vanessa Heinrich-Oliveira, José Maria Soares-Júnior, Edmund C. Baracat, Gustavo Arantes Rosa Maciel

**Affiliations:** Disciplina de Ginecologia, Departamento de Obstetrícia e Ginecologia, Hospital das Clínicas, Faculdade de Medicina da Universidade de São Paulo (HCFMUSP), São Paulo, SP, Brazil

## Abstract

• SARS-CoV-2 can temporarily reduce male fertility and alter sperm parameters.
• Reversible menstrual disturbances happened after infection and vaccination.
• IVF outcomes and laboratory parameters were not affected by SARS-CoV-2.

In 2020, the authors published a comprehensive review of the impact of COVID-19 on the human reproductive system. As described in the title, it was a changing perspective, due to the novelty of the disease, the velocity of the evolvement of the pandemic, and the extraordinary efforts to halt it.^1^ Given the latest updates on scientific publications about the impact of SARS-CoV-2 infection on human reproduction, the authors aimed to summarize the new available existing data related to the effects of COVID-19 on the human reproductive tract and aspects related to the disease and assisted reproductive therapy, pregnancy, and vaccination outcomes.

Both male and female reproductive tracts express ACE 2 receptor and TMPRSS2, although it is higher in men.^2^ Most recent studies, however, failed to detect SARS-CoV-2 presence in semen, follicular fluid, and vaginal secretion samples.^3^, ^4^, ^5^ Despite the absence of the virus in semen, impairment of seminal parameters is seen in men recovering from the disease, which could impair male fertility temporarily.^4^^,^^5^

Patel et al. have already demonstrated a higher susceptibility of the male gender to SARS-CoV-2 infection, as ACE2 expression is downregulated by estradiol levels and TMPRSS2 is upregulated by androgens.^6^ Fidecicchi et al. also suggested that estradiol plays a role in the modulation of innate immunity by suppressing the production of pro-inflammatory cytokines, interleukins and stimulating the production of anti-inflammatory cytokines. Higher levels of ACE2 ultimately lead to an increase in soluble ACE2 ectodomains in circulation, which serve as circulating scavengers for SARS-CoV-2, limiting their interaction with cell-bound ACE2.^7^

SARS-CoV-2 infection was associated with menstrual changes for a period up to three months^8^ in 16% of the women, especially those who experienced a greater number of COVID-19 symptoms. The most common changes were irregular menstruation, oligomenorrhea, and increased pre-menstrual syndrome.^9^ The endometrium also presents receptors that can be related to SARS-CoV-2 infectivity and change throughout the cycle, with higher expression in the secretory phase.^10^ A recent study confirmed these findings but showed that the maximum co-expression of ACE2 and TMPRSS2 was 0.73% of cells analyzed in the glandular epithelium during the early secretory phase. No co-expression was detected in the periconceptional period. Until now, it is suggested that the non-pregnant endometrium is at low risk for SARS-CoV-2 infection.^11^

Since the beginning of the COVID-19 outbreak, fertility societies kept publishing guidelines and recommendations worldwide. The initial suggestion was to discontinue all fertility treatments and encourage non-face-to-face assistance.^12^ However, the delay in carrying out fertility treatments can have serious consequences, particularly for patients with a diminished ovarian reserve and those facing gonadotoxic treatments.^13^ Studies also showed that universal screening in patients undergoing fertility treatment did not lead to more cycle cancellations nor did it affect gonadotropin doses, the number of oocytes and embryos cryopreserved, mature oocytes, and blast utilization rates.^14^

Pregnant women were considered at risk of severe SARS-CoV-2 infection and adverse maternal and neonatal outcomes. Cohort studies concluded that the infection tends to be more severe in the third trimester and in patients with comorbidities. Symptomatic cases most likely lead to increased rates of prematurity and intrapartum fetal distress than asymptomatic ones. Vertical transmission could not be completely ruled out yet, but neonatal infection rates appear to be low.^15^^,^^16^ A recent study by Chen et al. showed that cytotrophoblasts and syncytiotrophoblasts express various receptor-protease combinations and SARS-CoV-2 RNA can be detected in placental or membrane swabs from women infected with COVID-19. Thus, it can be assumed that vertical transmission is possible, although studies are still unclear. It is important to notice that the IgM assay is susceptible to false-positive or false-negative results, cross-reactivity, and additional testing challenges, making it difficult to diagnose congenital infections.^17^

Little is still known regarding perinatal outcomes of pregnancies resulting from assisted reproduction, as treatments were resumed in the last year. Engels Calvo et al. published a multicenter, prospective study of consecutive cases of SARS-CoV-2 infection in a pregnancy cohort comparing spontaneous pregnancies with those resulting from assisted reproduction treatment with either own or donated oocytes. There were no differences in the severity of SARS-CoV-2 infection between groups. Regarding perinatal outcomes, operative delivery was higher in the IVF group although cesarean section rates because of COVID-19 severe disease were similar between groups. IVF mothers experienced significantly more gestational hypertensive disorders regardless of the origin of the oocytes and had a higher risk of ICU admission, which was associated with preeclampsia and SARS-CoV-2 infection clinical presentation. Thromboembolic and hemorrhagic events, stillbirth, maternal mortality, and neonatal outcomes were similar for both groups.^18^

The search for mitigation of the pandemic provided the development of immunization in a surprisingly short time. However, several anti-vaccine groups developed a series of theories for the negative effects of the vaccine, including in relation to fertility, using unrealistic information. The influence of vaccines with mRNA showed no change in the parameters studied in relation to gametes and embryos. The success rates of fertilization treatments, as well as the functions of the theca and granulosa layer cells, were shown similarly in the comparison of women cured of COVID-19 infection, immunized and non-immunized.^4^ Orvieto et al. compared couples undergoing in vitro fertilization with or without immunization and showed stimulus, ovular and embryonic quality parameters were similar between groups.^19^ Mohr-Sasson et al. prospectively studied women who would undergo immunization by Pfizer-BioNTech COVID-19 in relation to ovarian reserve and performed the dosage of Anti-Mullerian Hormone (AMH) before and three months after its use. No difference was found in the study parameters, even after stratifying the women by age group.^20^

Data on SARS-CoV-2 infection is constantly changing and has been updated since the beginning of the pandemic. The most recent data regarding human reproduction shows there is no consistent evidence to support that COVID-19 affects ovarian tissue, and folliculogenesis nor that it can be considered a sexually transmitted infection. Vaccination proved to be safe and is indicated to couples undergoing assisted reproductive treatment, with no harmful in vitro fertilization outcomes.

## Conflicts of interest

The authors declare no conflicts of interest.
